# Tuberculosis of Talonavicular Joint Without Pulmonary Involvement in an Indian Child: A Report of a Rare Case

**DOI:** 10.7759/cureus.39707

**Published:** 2023-05-30

**Authors:** Sankalp Yadav, Gautam Rawal, Madhan Jeyaraman

**Affiliations:** 1 Medicine, Shri Madan Lal Khurana Chest Clinic, New Delhi, IND; 2 Respiratory Medicine, Max Superspeciality Center, New Delhi, IND; 3 Orthopaedics, ACS Medical College and Hospital, Dr. MGR (Maruthur Gopalan Ramachandran) Educational and Research Institute, Chennai, IND

**Keywords:** genexpert mycobacterium tuberculosis/rifampin (mtb/rif) assay, rifampicin, young child, tuberculosis, talonavicular joint

## Abstract

Osteoarticular tuberculosis is a rare entity, even in endemic countries. Isolated cases of tuberculosis of the talonavicular joint are sparse. Primary involvement of the talonavicular joint without pulmonary infection with *Mycobacterium tuberculosis* is rarest of rare. Here, we report a case of primary talonavicular joint tuberculosis without pulmonary involvement in an Indian child. To the best knowledge of the authors, it is the third such case ever reported in a child in the world. The patient presented with complaints of pain and swelling of the right foot. Detailed laboratory work-up backed by radiological investigations helped in establishing the diagnosis. He was managed conservatively with antitubercular chemotherapy with improvement in his symptoms and was transferred out to his native village.

## Introduction

Tuberculosis is a disease with a direct impact on the public health [1]. It is common in countries in Asia, Africa, and Europe [1]. Osteoarticular tuberculosis is a rare form of tuberculosis [2]. It constitutes 1-3% of all tuberculosis cases [2]. It is an extrapulmonary tuberculosis with a 15% share in total extrapulmonary tuberculosis cases [3]. Osteoarticular tuberculosis is mainly found in the spine and the weight-bearing joints, and is relatively rare in ankle and foot bones [3,4].

Tuberculosis of bone and joints of the foot are not associated with specific clinical features and therefore are difficult to diagnose [5]. Often such cases are diagnosed very late, thereby having a direct impact on patient management and treatment outcomes [3,5].

There is a paucity of data regarding tuberculosis of the talonavicular joint in children and to the best of our knowledge, only two such cases are available in the literature [6,7]. We present the third case, that of a 12-year-old Indian child who presented with complaints of pain and swelling in his right foot, which was diagnosed as tuberculosis of the talonavicular joint and was managed with antitubercular drugs.

## Case presentation

A 12-year-old Indian male was brought to the outpatient department by his parents with complaints of pain and swelling in the dorsum of the right foot for five months. He was unable to bear weight on the affected foot but there was no limp. The swelling was insidious in onset and gradually progressed with pain. The pain was continuous, generalized over the right foot, and aggravated on walking. His pain subsided a little after taking over-the-counter nonsteroidal anti-inflammatory drugs (NSAIDs). There was no history of cough, fever, night sweats, or weight loss. And there was no history of trauma. Besides, there was no history of tuberculosis in him or his contacts.

General examination revealed a young child with a temperature of 37 degrees Celsius, pulse of 81 per minute, blood pressure of 110/78 mm of Hg, respiratory rate of 19 per minute, and oxygen saturation (SpO2) of 99% on room air.

Local examination revealed a 3 x 3 cm swelling over the anteromedial side of the dorsum of the right foot with a smooth surface and tenderness on deep pressure over the talonavicular joint. The swelling was generalized with raised local temperature over the skin and did not subside on limb elevation. However, there were no dilated veins or discharging sinuses over it. The eversion and inversion movements of the right foot were slightly restricted and painful while dorsiflexion and plantar flexion were terminally painful; however, a full range of movement of the foot was present. Further, there was no clubbing, cyanosis, icterus, pallor, koilonychia, or lymphadenopathy. Systemic examination was within normal limits.

A probable diagnosis of tuberculosis of the foot was made with differentials of pyogenic osteomyelitis, fungal osteomyelitis, bone tumor, and granulomatous diseases such as gout, sarcoidosis, and amyloidosis.

He underwent an exhaustive radiographic and lab work-up with a raised erythrocyte sedimentation rate of 60 mm during the first hour with normal blood counts. The C-reactive protein value was 9 mg/l. His HIV test was non-reactive. A rheumatoid factor test was negative. The joint fluid test was negative for urate crystals.

**Figure 1:**
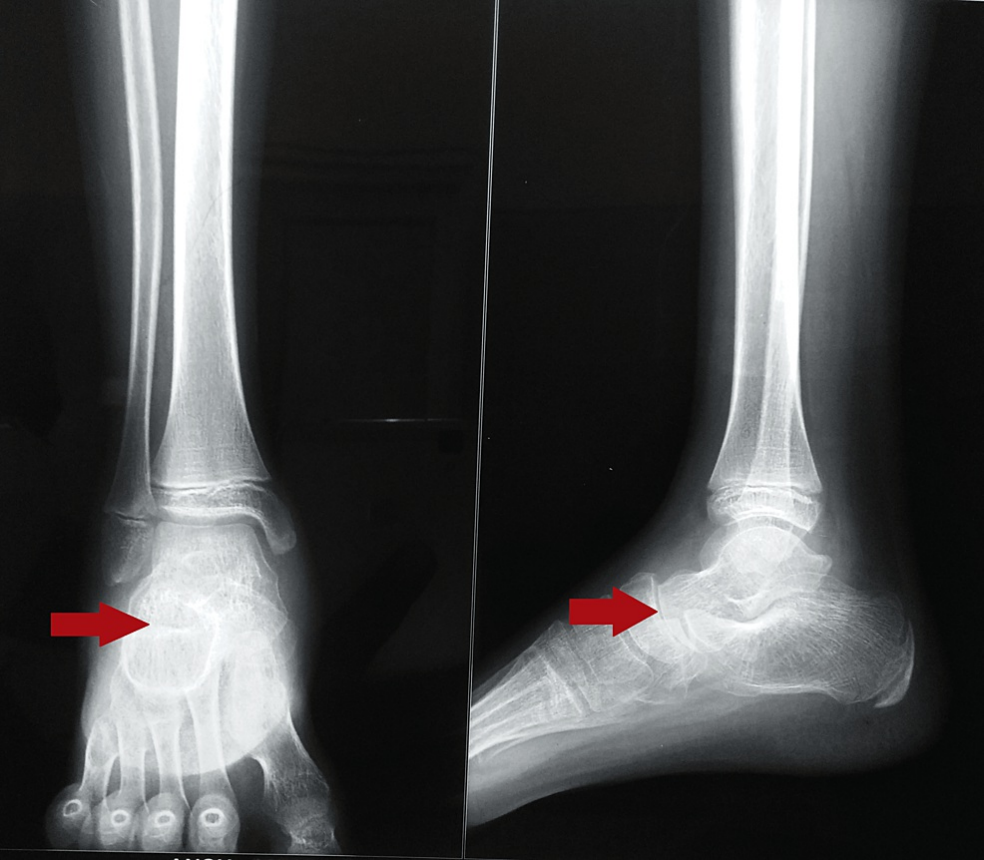
Radiograph of the right foot (anteroposterior and oblique views) showing gross talonavicular joint space narrowing

**Figure 2:**
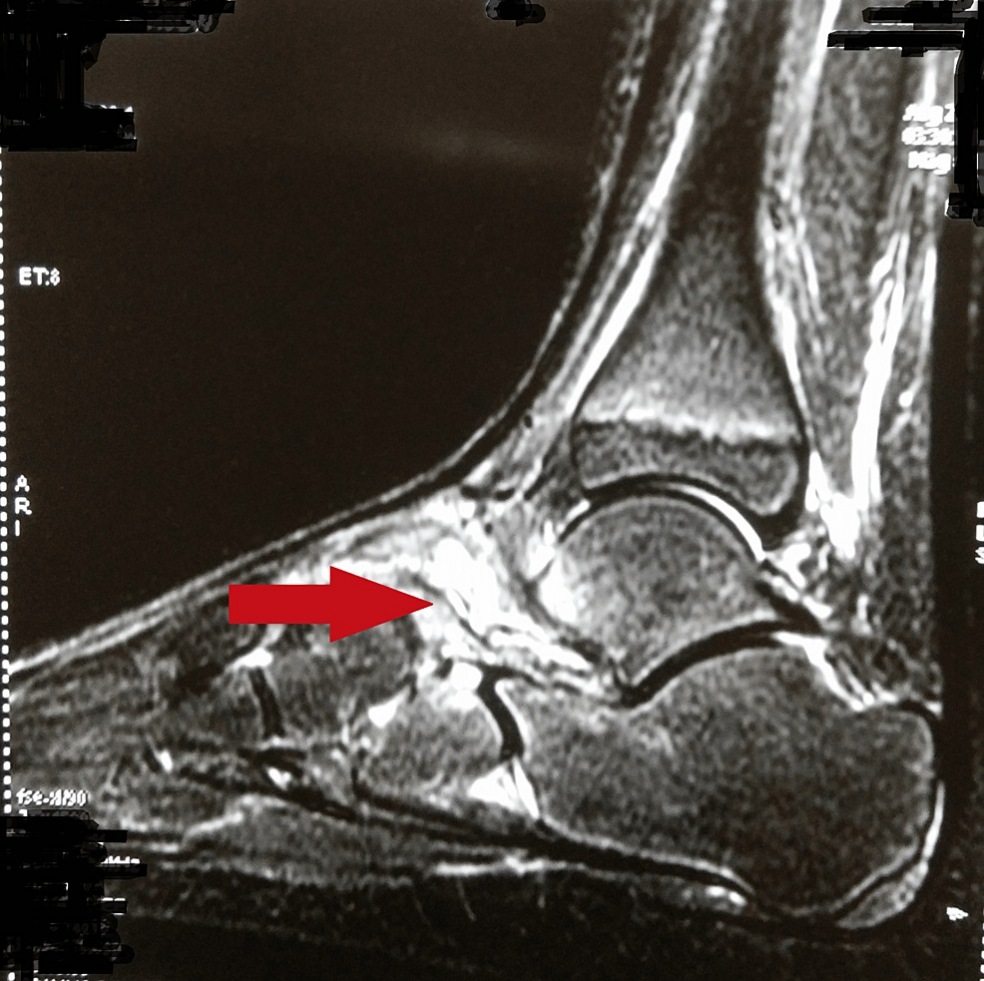
A magnetic resonance imaging of the right ankle suggestive of talus and navicular bones involvement with periarticular soft tissue collections

The radiograph of the right foot anteroposterior and oblique views showed gross talonavicular joint space narrowing (Figure 1). A chest radiograph was normal. A magnetic resonance imaging (MRI) of the right ankle was suggestive of periarticular subcutaneous and myofascial edema/synovial effusions with marrow edema involving talus and navicular bones with periarticular soft tissue collections (Figure 2). Minimal intertarsal effusion was noted in the talonavicular joint.

A fine needle aspiration cytology of the swelling was done. The aspirated fluid was suggestive of acid-fast bacilli on Ziehl-Neelsen staining. Histopathology was suggestive of tuberculosis with epitheloid granulomas with caseating necrosis. Further, the cartridge-based nucleic acid amplification test of the aspirated fluid revealed *Mycobacterium tuberculosis* detected (low) with no resistance to rifampicin. One more sample was sent for line probe assay (LPA) and culture to the National Reference Laboratory and the results were suggestive of *Mycobacterium tuberculosis* detected on LPA and grew on liquid culture system, Bactec Automated Blood Culture System (Becton, Dickinson and Company, Franklin Lakes, New Jersey, United States) with no resistance to any of the first-line antitubercular drugs.

Based on the radiographic and laboratory work, he was diagnosed as a case of primary talonavicular joint tuberculosis without pulmonary involvement and was initiated on antitubercular chemotherapy as per his weight initially with four drugs (for eight weeks) and followed with three drugs for a period of 10 months (40 weeks) (Table 1).

**Table 1:** Antitubercular treatment advised to the patient

Drug	Dose	Duration
Intensive Phase		
Rifampicin	450 mg	Eight weeks
Pyrazinamide	1000 mg	Eight weeks
Ethambutol	600 mg	Eight weeks
Isoniazid	300 mg	Eight weeks
Continuation Phase		
Rifampicin	450 mg	Forty weeks
Ethambutol	600 mg	Forty weeks
Isoniazid	300 mg	Forty weeks

**Figure 3:**
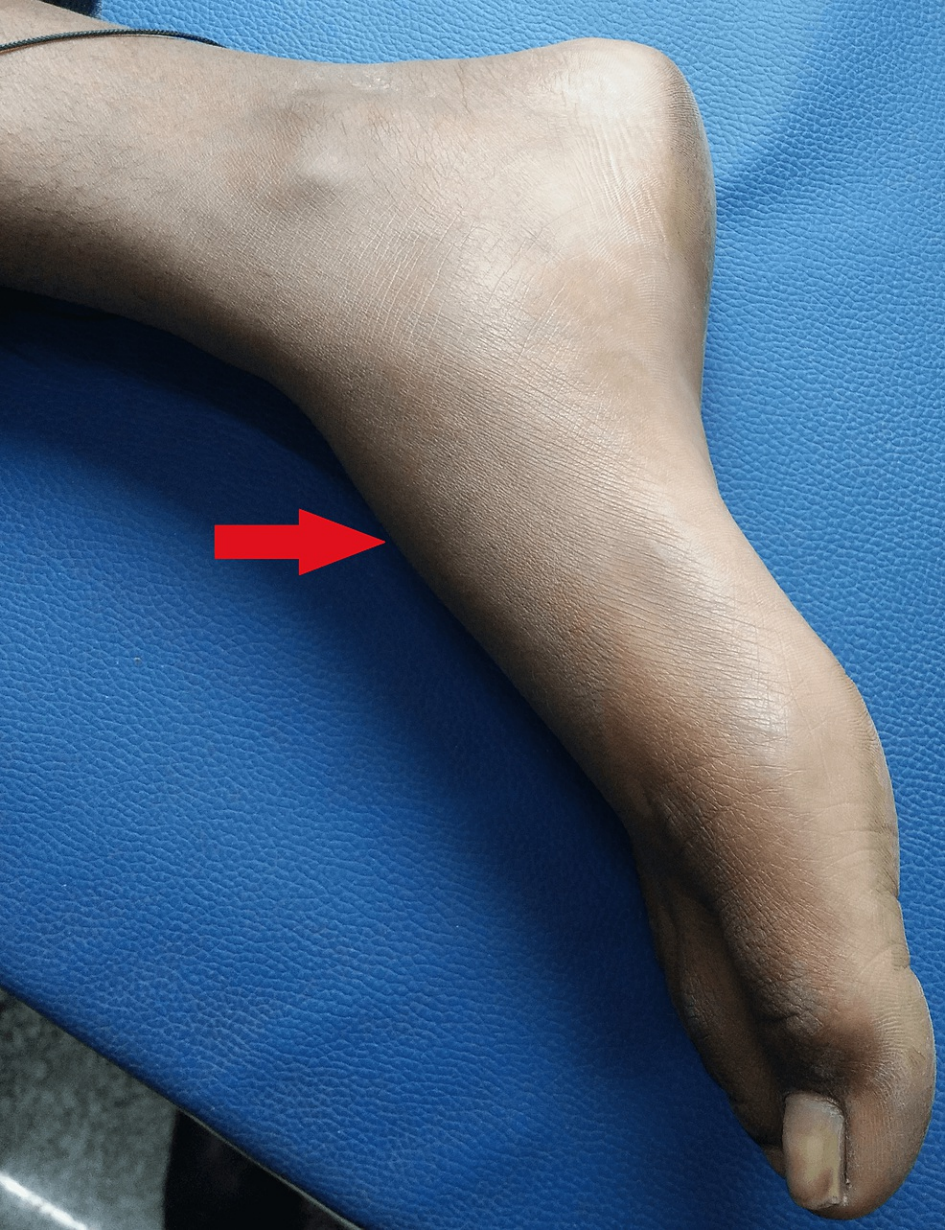
Gross image of right foot showing that the swelling had subsided

Along with this, he was given a tablet of pyridoxine 40 mg for the entire duration of treatment and was advised a high protein diet. He responded well to the treatment with no adverse drug reactions and after completion of seven months of treatment, on his request, he was transferred out to his native village. His swelling had subsided and there were no major complaints (Figure 3). We tried to contact him after his treatment completion but we could not retrieve his latest radiographs. 

## Discussion

A significant proportion of the world’s population is affected by tuberculosis and in high-burden countries, tuberculosis of bone and joints is often reported [8]. Osteoarticular tuberculosis of the foot is less common and infrequently reported [9]. This type of tuberculosis is mainly noted in the bones like the calcaneum, talus, first metatarsal, navicular, and medial and intermediate cuneiforms [9]. Isolated cases of tuberculosis of joints of the foot in children with no pulmonary involvement are very rare [6,7].

Clinical features are indistinguishable from other musculoskeletal disorders and usually seen as pain, stiffness, and swelling with a few reports of early muscle atrophy also available [9].

Osteoarticular tuberculosis is a difficult diagnosis [9]. The main reason for this is the paucibacillary nature of the disease [5]. As a result, it is an arduous task to establish the diagnosis with isolation of the bacteria [5,9]. Therefore, investigations like MRI or computed tomography (CT) must be used early if there is suspicion on a radiograph. Often, such patients present late, and thus prompt diagnosis backed with good clinical examination and laboratory work-up is essential [10].

A case similar to ours was reported by Birjandinejad et al. in 2012 [7]. The present case shares similarities with their case in no history of trauma, right foot involvement, clinical features, and absence of classical symptoms of tuberculosis like cough, fever, and other constitutional symptoms, and a normal chest radiograph [7]. However, our case differs from their case in age, absence of ulcer over the foot, availability of MRI reports, isolation of *Mycobacterium tuberculosis* on culture, and normal C-reactive protein levels. Besides, unlike their case, there was no surgical intervention required in our case [7].

Another case similar to ours was documented by Faizan et al. in 2017 [6]. Both cases share similarities in clinical features, absence of trauma history, absence of classical symptoms of tuberculosis like cough, fever, and other constitutional symptoms, and a normal chest radiograph [6]. Our case also shared similar radiograph findings with their case with narrowing of joint space, a raised erythrocyte sedimentation rate, and growth of *Mycobacterium tuberculosis *on culture. Further, no surgical intervention was done in the present case and in their case. However, our case differs from their case in age, side of the foot involved, availability of MRI reports, and normal C-reactive protein levels.

The management of osteoarticular tuberculosis of the foot is mainly conservative [11]. The use of antitubercular drugs is mentioned in the guidelines of the National Tuberculosis Elimination Program (India) [12]. However, in some cases, surgical interventions are required, which range from biopsy, synovectomy, and debridement, to joint-saving methods like a distraction in early cases, and arthrodesis of hindfoot joints and the ankle in advanced disease with joint destruction [9,11].

Due to the paucity of literature related to the present case, it is recommended that large-scale studies related to talonavicular joint tuberculosis from heavy centers be made available from the high-burden countries. 

## Conclusions

Osteoaticular tuberculosis of the talonavicular joint is a difficult-to-diagnose entity. The non-specific clinical features, paucibacillary nature of the disease, and paucity of literature, especially in children, even in high-burden countries are reasons for delayed presentations, diagnosis, and management. The treating clinicians should have a high index of suspicion to diagnose and manage such cases. Prompt diagnosis with histopathology and the use of imaging techniques like CT and MRI would help in reducing the unwanted delay in management, thereby preventing adverse consequences on the outcome for patients.
